# A Framework of Major Tumor-Promoting Signal Transduction Pathways Implicated in Melanoma-Fibroblast Dialogue

**DOI:** 10.3390/cancers12113400

**Published:** 2020-11-17

**Authors:** Barbara Bellei, Emilia Migliano, Mauro Picardo

**Affiliations:** 1 Laboratory of Cutaneous Physiopathology and Integrated Center of Metabolomics Research, San Gallicano Dermatological Institute, IRCCS, 00144 Rome, Italy; mauro.picardo@ifo.gov.it; 2 Department of Plastic and Regenerative Surgery, San Gallicano Dermatological Institute, IRCCS, 00144 Rome, Italy; emilia.migliano@ifo.gov.it

**Keywords:** melanoma, cancer associated fibroblast, tumor microenvironment, melanomagenesis

## Abstract

**Simple Summary:**

Melanoma cells reside in a complex stromal microenvironment, which is a critical component of disease onset and progression. Mesenchymal or fibroblastic cell type are the most abundant cellular element of tumor stroma. Factors secreted by melanoma cells can activate non-malignant associated fibroblasts to become melanoma associate fibroblasts (MAFs). MAFs promote tumorigenic features by remodeling the extracellular matrix, supporting tumor cells proliferation, neo-angiogenesis and drug resistance. Additionally, environmental factors may contribute to the acquisition of pro-tumorigenic phenotype of fibroblasts. Overall, in melanoma, perturbed tissue homeostasis contributes to modulation of major oncogenic intracellular signaling pathways not only in tumor cells but also in neighboring cells. Thus, targeted molecular therapies need to be considered from the reciprocal point of view of melanoma and stromal cells.

**Abstract:**

The development of a modified stromal microenvironment in response to neoplastic onset is a common feature of many tumors including cutaneous melanoma. At all stages, melanoma cells are embedded in a complex tissue composed by extracellular matrix components and several different cell populations. Thus, melanomagenesis is not only driven by malignant melanocytes, but also by the altered communication between melanocytes and non-malignant cell populations, including fibroblasts, endothelial and immune cells. In particular, cancer-associated fibroblasts (CAFs), also referred as melanoma-associated fibroblasts (MAFs) in the case of melanoma, are the most abundant stromal cells and play a significant contextual role in melanoma initiation, progression and metastasis. As a result of dynamic intercellular molecular dialogue between tumor and the stroma, non-neoplastic cells gain specific phenotypes and functions that are pro-tumorigenic. Targeting MAFs is thus considered a promising avenue to improve melanoma therapy. Growing evidence demonstrates that aberrant regulation of oncogenic signaling is not restricted to transformed cells but also occurs in MAFs. However, in some cases, signaling pathways present opposite regulation in melanoma and surrounding area, suggesting that therapeutic strategies need to carefully consider the tumor–stroma equilibrium. In this novel review, we analyze four major signaling pathways implicated in melanomagenesis, TGF-β, MAPK, Wnt/β-catenin and Hyppo signaling, from the complementary point of view of tumor cells and the microenvironment.

## 1. Introduction

Melanoma represents approximatively 4% of skin cancer cases but is the deadliest one, corresponding to 80% of skin cancer deaths and about 1–2% of all cancer deaths [1,2]. Its incidence is rising in most countries of the western world [3]. The transformation of melanocytes into melanoma requires a burden of mutations that can be caused by both endogenous and exogenous cues [4,5,6]. However, genetic studies demonstrated that sporadic melanoma are associated to allele variants with high prevalence and low penetrance indicating that environmental factors play a key role in melanoma development [7,8,9]. Among them, the exposure to ultraviolet (UV) rays has a significant impact on skin biology and homeostasis [10]. The most deleterious effect of UV radiations is the direct damage to DNA. In addition, UVA not only contributes to the direct formation of DNA lesions but also impairs the removal of UV photoproducts from genomic DNA through oxidation and damage to DNA repair proteins [11,12]. In the epidermis, melanocytes, which are classified as intermittent mitotic cells [13] and normally divide only on demand, are more prone to accumulate damage than rapidly dividing cells such as keratinocytes. Likewise, melanocytes have a reduced repair capacity for oxidative DNA damage than skin fibroblasts [14]. Moreover, frequent excessive exposure to UV light impacts on melanocyte microenvironment within the epidermis, contributing to melanoma onset [15,16]. For example, alterations in the composition of basement membrane and dermal extracellular matrix might anticipate melanomagenesis, facilitating disease occurrence. In chronically sun-exposed skin, qualitative and quantitative alterations of dermal extracellular matrix proteins causing loss of tensile strength, increase fragility and impair wound healing [17]. The expression of type VII collagen that anchors fibrils at the dermal–epidermal junction by keratinocytes is decreased in UV-irradiated skin areas. UV-irradiated skin produces several enzymes such as matrix metalloproteinases (MMPs), which degrade dermal collagen fibers (especially type I collagen) and elastic fibers. This process causes an overall modification of mechanical properties of tissues that is part of the photoaging process [18,19]. Instead, in the contest of melanoma, MMPs altering the basement membrane and dermal ECM architecture can facilitate invasion of tumor cells. Accordingly, excessive production of MMP1, MMP2 and MMP9 has been frequently observed in melanoma patients [20,21,22]. Tissue surrounding benign nevi and melanomas display greater stiffness than normal skin indicating that mechanical properties of the matrix impact on melanoma initiation or progression [23]. Increasing evidence suggests that tissue rigidity or matrix stiffness controls phenotypic states and contributes to the invasive process in advanced melanoma [24]. Thus, in the skin, due to continuous extrinsic stimulation, persistent alteration in the extracellular matrix can act independently to tumor onset moving as a driver of the tumorigenic process. In addition, biological behavior of normal dermal and epidermal cells is constantly influenced by external agents. Since fibroblasts are long lived cells constantly undergoing damage accumulation, they are considered a relevant player of skin carcinogenesis. Following UV light irradiation, keratinocytes secrete melanocyte growth factor (including α-melanocytes stimulating hormone, α-MSH and endothelin-1, EDN1), which increase cytokine and the melanin production and transfer preventing further damage caused by UV [25,26,27]. In vitro, UVA and UVB activate bFGF production by both skin fibroblasts and keratinocytes [28]. Keratinocytes additionally increase the secretion of hepatocyte growth factor (HGF) and transforming growth factor-β1 (TGF-β1) inducing proliferation in melanocytes that in turn become more susceptible to transformation [29].

Transitory activation of melanocytes is part of the tanning response, the main physiological process protecting the skin from UV light. However, aberrant increased production of growth factors is considered part of the acquisition of stress-induced premature senescent phenotype and the corresponding senescence-associated secretory phenotype (SASP) as those promoted by chronic sun exposure. Correspondingly, growth factors hyperproducing senescent fibroblasts are frequently described in age spot and melasma, two pathological condition characterized by hyperpigmentation [30,31,32,33]. It is extensively documented that senescent fibroblasts accumulate in habitually sun-exposed skin and orchestrate stroma modification into a tumor-promoting one [34,35,36,37]. In line with other cancer types, the secretory profile of melanoma-associated fibroblasts largely overlaps with that of senescent fibroblasts [38,39]. Properly, multivariate analyses demonstrate increasing age is the strongest independent adverse prognostic factor together with Breslow thickness [40]. Tumor cells utilize fibroblast-secreted growth factors to facilitate their own survival and proliferation. The secretory profile of fibroblasts is also critical in metabolic and immune reprogramming of the tumor microenvironment with an impact on angiogenesis regulation and adaptive resistance to therapy [41,42,43,44]. Due to the intense melanoma-stoma crosstalk, fibroblasts progressively modify their biological feature, presenting a molecular signature that partially overlaps with the cancer one.

This review focuses on major intracellular signaling pathways deregulated in melanoma analyzed from the reciprocal point of view of melanoma cells and MAFs.

## 2. Transformation of Normal Fibroblasts to Melanoma-Associated Fibroblasts

Physiologically, melanocytes reside within the basal layer of the epidermis and interplay tight contacts with epidermal keratinocytes through E- and P-cadherin adhesion proteins, whereas an intense communication networks mediated by soluble factors explains the deep influence of dermal compartment in melanocyte biology [45,46,47]. However, during melanoma progression, there is a progressive loss of E-cadherin [48,49,50] and gain of N-cadherin [49,51,52,53], which not only frees melanoma cells from control by keratinocytes, but also provides new adhesion characteristics [54,55,56]. Consequently, melanoma cells acquire invasive properties, violate the basement membrane and invade the underlying dermis establishing unusual homotypic interaction between melanoma cells and heterotypic cell–cell contact with fibroblasts, endothelial and immunocompetent cells. All these elements synergistically play a specific role in disease progression. Cadherin switch as well as integrins expression profile modification also implies the activation of inappropriate survival signals [57,58] and thus enhances the malignant phenotype [51,55,59].

**Figure 1:**
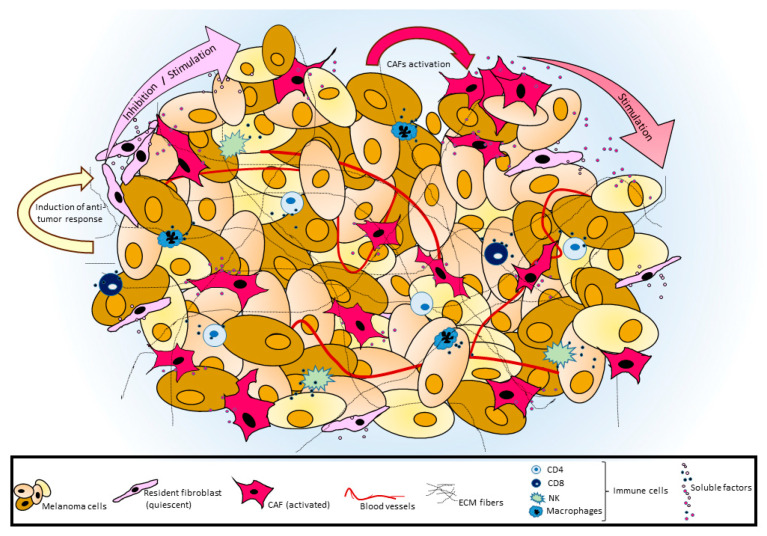
Schematic representation of tumor–stroma cross-talk. In melanoma, tumor cells share their microenvironment with MAFs, immune cells and blood vessels. Resident fibroblasts might oppose an early anti-tumor activity or facilitate tumor development, whereas, during disease progression, activated MAFs gradually acquire a marked pro-tumorigenic phenotype. An intense bi-directional exchange of soluble factors between melanoma and surrounding cells significantly modifies in MAFs several intracellular signaling pathways including oncogenic pathways. Extracellular matrix supports tumor architecture and influences various signal transduction pathways in both tumor and associated cells.

Melanoma cells actively interact with stromal cells, not only through direct cell–cell but also through cell–matrix interactions and secreted growth factors and cytokines. A complex network of soluble bioactive molecules contributes to the alteration of the host tissue and to the definition of malignant behavior of melanoma. An exclusive feature distinguishing melanoma from other tumors is the communication through melanosome, tissue-specific organelles deputies to the extracellular melanin distribution [60]. Fibroblasts around melanoma contain in their cytoplasm a significantly higher density of melanosome [61]. Melanosomes released from melanoma cells carrying pro-inflammatory molecule and several microRNA are able to transform dermal fibroblasts into pro-tumorigenic [61]. Evidence that fibroblasts begin to aggregate in the dermis at early stages of melanoma initiation, before melanoma cells invade, underlies the importance of paracrine communication between melanoma cells and surrounding. Thus, starting from early tumor stage, due to continuous paracrine stimulation by transformed cells, surrounding stromal fibroblasts are induced to initiate phenotypic, molecular and biochemical transitions and to transdifferentiate into cancer-associated fibroblasts (CAFs). Cancer-associated fibroblasts represent one of the major players in tumor–stroma network. CAFs acquire myofibroblast features and produce several growth factors that contribute to tumor cells proliferation, survival and metastasis [42,62]. CAFs are similar to myofibroblasts present during wound healing or the fibrotic conditions. In fact, desmoplastic wound healing-like tumor stroma is frequently referred as a consequence of mutual interaction of tumor cells and CAFs [63]. CAFs are distinguished from their normal counterparts by the expression of several markers such as alpha-smoot muscle actin (α-SMA), fibroblast specific protein-1 (FSP-1 also referred as S100A4), fibroblast-activating protein (FAP), platelet derived growth factor receptor-alpha/beta (PDGR α/β), tenascin-C, collagen 11-α1 (COL11A1), vimentin and fibronectin. However, a univocal molecular definition of CAFs profile is yet lacking. Clinically, the presence of many myofibroblasts in the tumor microenvironment has been associated with elevated risk of invasion, metastasis and a poor prognosis [34,64]. In addition to resident fibroblasts, there are several sources of CAFs, including bone marrow mesenchymal cells and endothelial cells [62,65]. In the case of melanoma, due to the prevalent localization at the junction of the epidermis and the dermis, dermal fibroblasts are considered the major source of CAFs, also referred to as melanoma-associated fibroblasts (MAFs) [42,66,67,68] (Figure 1). MAFs are less frequent compared to CAFs in other solid tumors [66]. Fibroblasts are associated to melanoma cells at all stages of disease and their functional contribution to disease progression has been largely documented but now increasing data also highlight their antitumor actions [69,70]. As an extreme consequence of the intimate relationship between melanoma and fibroblast, an original study described cell fusion events capable of generating tumor–stroma cell hybrid clones [44]. It is not fully clear if the dual nature of cancer microenvironment reflects the contemporary presence of heterogenic populations or if differences reside in disease evolution. Since MAFs co-evolve with tumorigenic cells it is possible that an early anti-tumor phenotype is replaced by a pro-tumorigenic one during disease progression. In line with the idea that MAFs co-evolve with melanoma cells, several research papers demonstrated an inhibitory function of dermal fibroblasts during tumor onset. This is largely because normal dermal fibroblasts, which are mostly quiescent cells in healthy condition, function as controller of tissue homeostasis. In vitro, co-culture experiments using normal fibroblasts and cells isolated from primary melanoma evidenced repressive influence on melanoma cells [71]. Multiple factors have been implicated in the transition of normal tumor-suppressive fibroblasts into reactive and tumor-promoting CAFs [72].

One of the biological behaviors of activated fibroblasts is increased proliferation rate [73]. A possible explanation of augmented proliferation of stromal cells might reside in the pro-mitogeic tumor milieu. At least during early stage of disease, the simple nearness to transformed cells could explain the involvement of non-cancerous cells that endure the extraordinary tumor secretory activity of melanoma cells. Proteomic analysis revealed that factors released in the culture medium by melanoma cells stimulate in dermal fibroblasts a general increase in protein synthesis. In particular, the biological process involved in fibroblast reprograming are those related to mRNA catabolic process, translation initiation, protein targeting to membrane and synthesis of metabolic-related small molecules [74,75]. Paired analysis of melanoma cells and associated MAFs revealed a trend of functionally coordinated reorganization of metabolic pathways, cytoskeleton reorganization and ECM remodeling [74]. Fibroblast reprograming is a crucial step for melanoma progression as demonstrated by the correlation between capacity of melanoma cells to alter fibroblast gene expression and the invasive potential in vitro [76]. Specifically, metastatic melanoma increases the production of cytokines and chemokines by MAFs more efficiently than nonmetastatic melanoma [76]. Among the immunomodulators involved in MAFs activation, IL1β seems to be a driver of melanoma invasion both in vitro and in vivo [76]. Cytokines produced by dermal fibroblasts, such as interleukin-6 and -8 (IL-6 and IL-8), interferon gamma (INFγ), tumor necrosis factor-alpha (TNF-α) [77,78] and a variety of CXCLs [37], have the capacity to mobilize immune cells. The balance between pro-inflammatory and anti-inflammatory cytokines in tumor area strongly impact on patient’s prognosis. Cytokines profile supporting M1 macrophage differentiation sustain CD4+ and CD8+ T-cells infiltrating the tumor microenvironment and favorable clinical outcome, whereas alternative M2 macrophage polarization present immune-suppressive function. Since MAFs are the main producer of suppressive cytokines [46], their role in immune escape and tumor progression is relevant. MAFs suppress NK-cell activity and CD8+ cytotoxic activity [66,79]. Release of IL-8, CCL2/MCP1 (monocyte chemoattractant protein-1) and tissue inhibitor of metalloproteinase 2 (TIMP-2) by fibroblasts when co-cultured with melanoma cells has also been implicated in the angiogenic process indicating that recruitment of microvascular endothelial cells depends on the synergic melanoma-fibroblast network [80].

Considering all these pleiotropic roles, MAFs are considered a promising target for melanoma therapy. Essentially, targeting CAFs has been investigated by using agents aiming to eliminate or reprogram CAFs. However, in mouse model, reduced stromal content accelerates tumor growth and angiogenesis and full depletion of CAFs induces immunosuppression [81,82]. Thus, based on fibroblasts exceptional phenotypic plasticity, therapeutic re-orientation of CAFs to an anti-tumor phenotype seems to be more promising. Therefore, to efficiently reprogram CAFs’ activity against tumor, we need to exactly know the contribution of stromal fibroblasts in a specific tumor type and to promote exclusively anti-neoplastic properties.

## 3. Significance of Altered Intracellular Signal Transduction Pathways in Melanoma and Associated Fibroblasts

### 3.1. Wnt Signaling

Signal activation due to the Wnt family of secreted glycoproteins is involved in embryogenic development, cell polarity, tissue homeostasis and cell proliferation in adult stage [83,84]. β-catenin is the key protein regulating Wnt signaling-mediated gene expression [85]. Moreover, β-catenin is deeply implicated in cadherin-based cell adhesion [86,87]. In the absence of Wnt ligands, β-catenin is recruited into a destruction complex that contains adenomatous polyposis coli (APC) and AXIN, which facilitates the phosphorylation of β-catenin by casein kinase 1 (CK1) and then glycogen synthase kinase-3 beta (GSK3β) leading to its ubiquitylation and proteasomal degradation. Following the binding of Wnt factors to their receptors (frizzled, FZD and low-density lipoprotein receptor protein 5/6 (LPR5/6)), cytosolic GSK-3β is sequestered, and the phosphorylation of β-catenin is prevented. The blocking of β-catenin degradation leads to its stabilization in the cytosol and consequent translocation into the nucleus, where it binds to members of lymphoid enhancer-binding factor (LEF)/T-cell specific factor (TCF) family and some other co-regulators to promote the transcription of ubiquitous genes such as *Jun*, *c-Myc* and *CyclinD-1*, most of which encode oncoproteins [88,89]. In addition, β-catenin is a co-activator for the expression of melanocyte-lineage restricted genes including Microphthalmia-associated Transcription Factor-M (*MITF-M*) [90,91,92], Dopachrome Tautomerase (*DCT*) [91,93,94,95] and *Brn-2* [96]. The ability of Wnt/β-catenin signaling to drive the expression of differentiation-related genes reflects its critical role in melanocyte development [91,97,98] and adult melanocyte stem cells mobilization [99,100]. Given peculiar involvement of β-catenin in both proliferation and differentiation of melanocytes, it is not surprising that in this type of cells its expression level is subjected to tight regulation. For example, in normal melanocytes in vitro, reduced β-catenin gene transcript by RNA interfering leads to a rapid stabilization of the corresponding protein capable of restoring the physiological level of expression [101].

As demonstrated in numerous studies, the aberrant activation of Wnt signaling contributes to malignant cell transformation and neoplastic proliferation with further metastatic dissemination and resistance to treatment [102,103,104,105]. Deregulations in the canonical Wnt signaling in cancer may result from gain-of-function gene alterations and epigenetic mechanisms. Nonetheless, β-catenin, *APC* and *AXIN2* mutations are rare in primary melanoma specimens [101,106,107,108,109,110,111], in comparison to well-characterized melanoma cell lines cultured in vitro for a long period [112]. This suggests that elevated β-catenin protein level confers proliferative advantage under a selective pressure such as in vitro cell growth. In melanoma, epigenetic regulation of Wnt antagonists such as Dickkopf proteins (DKKs), Wnt inhibitor factor-1 (WIF1) and soluble frizzled-related protein-2 sFRP2 contributes substantially to cell-autonomous activation of Wnt/β-catenin signaling [113,114]. *DKK2* and *DKK3* have been found upregulated in the good-prognosis melanoma patients presenting basal high immune signature [115]. Unlike most cancers, where Wnt signaling is considered a driver of both tumor formation and progression, in human melanoma, there are contradictory results [116,117,118]. The major discrepancies emerged from the comparison between studies performed with melanoma cell culture model and investigations based on immunohistochemical analyses in skin biopsy and clinical outcome. In vitro studies proposed that an increased nuclear translocation and activity of β-catenin promote melanoma proliferation [119] and invasion [105]. By contrast, diverse studies linked the activation of Wnt/β-catenin signaling to decreased proliferation [120] and repressed invasion [121] and migration [122]. In vivo observations from melanoma patients indicated that nuclear β-catenin correlates with improved survival [120,121,122,123]. Furthermore, almost all benign nevi are positive for nuclear β-catenin [124,125]. Adding complexity to this scenario, changes in Wnt signaling pathway have been linked to phenotype switching of melanoma cells between a highly proliferative/non-invasive (high β-catenin expressing cells) and a slow proliferative/metastatic (low β-catenin expressing cells) condition [126,127,128,129]. These data collectively suggest that the ambiguous role of Wnt pathway activation in melanoma strongly depends on the combination of both intracellular and microenvironmental contexts. The expression and transcriptional activity of β-catenin inversely correlate to immune activation and it has been proposed as a predictive marker of immunotherapy response [115,116,130,131,132,133,134]. The mechanism by which β-catenin promotes resistance to immunotherapy involves the reduced secretion of attractant chemokines that allows impaired infiltration and activation of dendritic and T cells [130]. Conversely, MAPK inhibitors demonstrated an enhanced efficacy in cultured melanoma cell lines with activated Wnt/β-catenin signaling [135,136]. However, in line with the idea that patient’s immune activity contributes to MAPK inhibitors outcome, in vivo studies did not confirmed improvements in patient’s survival presenting intrinsic β-catenin activation [137].

In the tumor background, in addition to genetic and epigenetic mechanisms, increased paracrine factors from the surrounding tissue might contribute for the activation of Wnt signaling in tumor cells [138]. Among these, Wnt ligands and several growth factors are frequently hyperproduced by CAFs [139,140] such as hepatocyte growth factor (HGF) [141] and platelet-derived growth factor (PDGF) [142]. It has been proposed that elevated level of sFRP2, a Wnt antagonist, secreted by aged fibroblasts could facilitate the acquisition of a metastatic, therapy-resistant state of melanoma [143]. Modification of Wnt pathway modulators in melanoma cells, including Wnt5a [128,144,145], Wnt7b, Wnt10b [146], Frizzled-3 (FZD3) [130] and DKKs [113] has been largely investigated as a cell autonomous mechanism responsible of signaling activation in tumor cells. However, this intense secretory activity might also deeply influence neighboring cell populations. Interestingly, Wnt ligands secreted by tumor cells could stimulate the polarization of tumor-associated macrophages (TAMs) to M2 tumor promoting subtype via Wnt signaling [147]. Thus, tumor paracrine activity might also play a relevant role in Wnt signaling regulation of other skin cell types, including mesenchymal cells. In dermal fibroblasts, transient stimulation of Wnt/β-catenin signaling is associated to an activated state of this type of cells during tissue repair [148,149,150,151]. Sustained Wnt/β-catenin activation in dermal fibroblasts is involved in pathogenesis of fibrotic diseases including hyperplastic wounds [152] and keloids [153,154,155]. Stroma recruited by melanoma resembles fibrotic microenvironment of persistent wound healing, since the entire tissue experiences a chronic injury due to the damage caused by tumor growth. As with other cancers, nuclear and cytoplasmic β-catenin has been demonstrated highly expressed in MAFs located around and in the melanoma tissue [72]. In 3D multicellular tumor spheroid model and in in vivo mouse melanoma model, skin fibroblasts ablated for *β-catenin* gene demonstrated reduced ability to support the growth of B16F10 melanoma cells [70,72]. In this case, the observed reduced number of stromal fibroblasts partially explain the diminished inhibitory effect of dermal fibroblast on melanoma formation. However, deactivation of β-catenin also weakens the expression of HGF and ECM proteins in residual fibroblasts [72,156]. Interestingly, β-catenin loss in fibroblast affects the RAF-MEK-ERK signaling cascade suppressing melanoma cell proliferation and cells death at the same time [72,157]. Shao and co-workers reported that the expression of Wnt-induced secreted protein-1 (WISP-1), a β-catenin target gene, is almost undetectable in areas with melanoma and in the surrounding tissue, whereas strong expression has been observed in non-activated fibroblasts of uninvolved skin [158] indicating a negative correlation between WISP-1 expression and a permissive tumor microenvironment. However, in this case, deregulated WISP-1 expression has been linked to the suppression of Notch signaling in MAFs rather than to the activity of Wnt pathway. As observed for other mesenchymal cells, independently of the presence of Wnts ligand at the extracellular level, Wnt signaling could be coordinately modulated in melanoma and stromal cells also by tissue stiffness [159]. Activation Wnt/β-catenin signaling in response to change in substrate stiffness might be stabilized or reinforced by a positive feedback loop based on the transcription of the β-catenin target gene *wnt-1* [159].

### 3.2. Hippo Signaling

Hippo signaling is an important regulator of cell proliferation and survival in animals playing a critical role in organ size control, stem cells homeostasis, cell polarity and shape [160,161]. The Hippo pathway is regulated upstream by extracellular mechanosensory signals arising from perturbation of actin cytoskeleton and adhesion change, as well as by a variety of extracellular signaling molecules. Hippo activity is deregulated in many cancers, despite mutations of pathway components are uncommon especially in melanoma [162,163]. Alteration of gene copy number among Hippo pathway elements have been frequently observed [164]. The Hippo signaling pathway includes a kinase cascade that modulates different proteins in order to phosphorylate and inactivate its main downstream cytosolic effectors, yes-associated protein (YAP) and tafazzin (TAZ), which direct gene expression via control of the Transcriptional enhancer factor (TEAD) family of transcription factors [165,166]. Nuclear YAP/TAZ interact with several important transcription factor including TCF/LEF, small mother against decapentaplegic factors (SMADs), cAMP response element-binding protein (CREB), myoblast determination protein 1 (MyoD) and tumor protein p73 (TP73) controlling cell proliferation and apoptosis [167,168,169]. This pathway is thought to be central to uveal melanomagenesis as YAP is hyperactive in uveal melanoma cells and mediates the oncogenic effect of guanine nucleotide binding protein (G protein) q polypeptide (GNAQ), or G protein α 11 (GNA11) mutations, which occur in approximately 80% of these type of melanoma [170,171]. YAP protein expression is elevated in most benign nevi and primary cutaneous melanomas but present at only very low levels in normal melanocytes [163]. Furthermore, since Hippo pathway is modulated by adhesion change and mechanical signaling, there is a strong possibility that extracellular stimuli from the melanoma microenvironment such as ECM modification might therefore impact on its activation. In melanoma patients, increased collagen and fibronectin abundance correlates with YAP nuclear localization [24]. Further analysis of the same patient cohort evidenced that melanoma cells positive for YAP nuclear staining present elevated MITF expression and a proliferation/differentiation signature. Proliferation and differentiation are not mutually exclusive events in the melanocyte lineage and are both promoted by MITF. By contrast, low level of MITF are associated to a dedifferentiated/invasive phenotype [126,172]. Several other studies indicated that hyperactive YAP is sufficient to drive cells switch from proliferative to invasive phenotype [173,174]. In murine xenograft model silencing the expression of YAP and TAZ results in reduced proliferation of human melanoma cell lines and decreases lung metastasis [133]. The E-cadherin/catenin complex functions as an upstream regulator of the Hippo signaling combining loss of E-cadherin at the cell surface with β-catenin and YAP nuclear accumulation [175]. Added evidence demonstrated cross-regulation between Wnt/β-catenin and Hippo signaling: TAZ is targeted for degradation by the β-catenin destruction complex [176,177]. On the other hand, YAP and TAZ can retain β-catenin in cytoplasm limiting Wnt/β-catenin signaling [178]. Thus, YAP and TAZ can be viewed as integral components of the Wnt/β-catenin signaling pathway in addition to their role in Hippo signaling [178,179]. Interestingly, TGF-β produced by stromal fibroblasts might exert a fine regulation of YAP transcriptional activity promoting YAP/SMAD interaction instead of YAP/PAX3 transcription complex redirecting cells to a less differentiated more aggressive state similar to melanocyte stem cells [24].

In cutaneous melanoma, evidence from cell lines supports not only a role of YAP/TAZ in cell invasion but also in resistance to target therapy and immunotherapy [171,180,181]. YAP promotes PD-1 expression driving immune evasion in BRAF inhibitors-resistant melanoma [181,182]. However, opposite to tumor cells, activation of YAP and TAZ in CAF exerts a tumor-suppressive function. In fact, deletion of *YAP* and *TAZ* in these peritumoral cells accelerated primary liver tumor growth. Experimental hyperactivation of YAP in peritumoral hepatocytes triggered regression of melanoma-derived liver metastasis. Since tumor cell survival depend on the relative activity of YAP and TAZ tumor and surrounding tissue, it has been hypothesized that the major function of YAP/TAZ in tumor cells is to elevate their competitive fitness and to “protect” them from the tumor-suppressive action of the surrounding parenchyma [182]. According to the concept of cell competition, an interesting point resides in the concept that it is the relative level and not the absolute level of a molecular pathway (not only Hippo pathway) that determines which cells (peritumoral or tumoral) lose competition. On the other hand, acquired mutations render cancer cells more competitive than non-neoplastic cells compromising tumor elimination [183].

### 3.3. TGF-β Signaling

The transforming growth factors (TGF)-β family of growth factors are secreted multifunctional cytokines that signals via plasma membrane TGF-β type I and type II receptors and intercellular small mother against decapentaplegic (SMADs) transcriptional effectors [184]. TGF-β controls tissue remodeling during embryonic development, angiogenesis, tissue repair and several cellular functions, such as cell growth, adhesion, recognition, cell fate determination and apoptosis [185,186]. In the skin, TGF-β is important for the wound healing process, especially in burn wounds [187]. TGF-β has a dual action in cancer as a tumor suppressor and a tumor promoter. As a tumor suppressor, it inhibits tumorigenesis by inducing growth arrest and apoptosis. As a tumor promoter, it induces tumor cell migration and stimulates epithelial to mesenchymal transition, a process during which cancer cells lose epithelial features and activate genes that increases cell motility and dissemination [35,36,188].

Alterations of TGF-β signaling, including loss-of-function mutations in genes encoding TGF-β receptors or SMAD proteins, confer escape from the antiproliferative activity of TGF-β [189]. Melanoma produces increasing amounts of TGF-β with disease progression [190,191], providing an optimal microenvironment for undisturbed tumor growth. Contrary to other tumor types, no genetic alteration of TGF-β signaling molecules has been identified in melanoma [192]. Among different forms, TGF-β1 is secreted by normal melanocytes and melanomas at various stages, while TGF-β2 and TGF-β3 levels rise early in melanoma and increase with tumor progression [193]. In addition, a correlation between TGF-β2 expression and tumor thickness has been reported [194]. Although melanoma cells efficiently respond to TGF-β at the receptor level, in contrast to normal melanocytes, melanoma cells display various degree of desensitization to the growth inhibitory activity of TGF-β. Resistance to the growth inhibitory activity of TGF-β has been explained by the frequent aberrant activation of MAPK pathway in melanoma that is capable to reprogram intracellular TGF-β cascade [192]. Similar to wound-healing process, tumor-derived TGF-β is likely to recruit other stromal cells. It is well demonstrated that transforming growth factor-β produced by tumor cells may promote tumor growth and advancement by modifying the microenvironment [56]. Forced overexpression of TGF-β1 by melanoma cells activates stromal fibroblasts, leading to augmented collagen, fibronectin, tenascin and α2 integrin expression. In experimental mouse model, tumors generated by subcutaneous co-injection of fibroblasts with melanoma cells demonstrated that TGF-β-overexpressing melanoma cells exhibit fewer necrotic and apoptotic cells and form more lung metastases than control melanoma cells [22]. Thus, activation of stromal fibroblasts by tumor-derived TGF-β provides an optimal microenvironment for tumor progression and metastasis [56,64]. Autocrine loop based on TGF-β and stromal derived factor 1 (SDF-1) is necessary for the consolidation of activated CAF phenotype [43,195]. Mechanical stress is the second major factor for myofibroblasts activation [64,196]. Initial small changes in tissue stiffness occur during the inflammatory response in tumor development and seem to be induced by increased collagen production and crosslinking [197,198]. Secondarily, stiff ECM promotes myofibroblast phenotypic conversion improving the efficiency of latent TGFβ1 activation [64]. Metastatic and primary melanoma cell lines overexpress collagen VI and in vivo the level of this type of collagen positively correlates to advanced stages [197,199]. Hyperactive TGF-β signaling associated to loss of caveolin-1 promotes tumorigenesis by shifting fibroblasts toward catabolic metabolism, a mechanism that generates energy-rich metabolites [43,200]. Another member of the TGF superfamily, Nodal, an embryonic morphogen not expressed in healthy adult tissues, has been demonstrated highly present in MAFs in vivo and in vitro [201,202]. Fibroblasts activated by Nodal, promote melanoma proliferation in vitro and in xenograft tumor models. A recent study demonstrated that the extremely high level of TGF-β1 produced by melanoma cells and detected in patients’ sera is also capable to activate fibroblasts of distant uninvolved skin [203]. This is of particular interest since recruitment of MAFs also supports the generation of metastatic niche necessary for invasion of distant organs [204]. Based on this observation, it is possible to extend, at least in advanced disease stage, the concept of melanoma microenvironment on the entire body.

### 3.4. MAPK Signaling

The mitogen-activated protein kinase (MAPK) pathway activation is a critical player in the biology of different types of cancer and is the most frequent pathway aberrantly activated in melanoma [205]. Up to 70% of melanomas exhibit activating mutations within the kinases *BRAF* gene [206,207,208,209] and approximately 15% of melanomas within *NRAS* gene [210,211], resulting in constitutive and sustained activation of downstream targets, RAS–MEK–ERK1/2 axis, in addition to unresponsive negative feedback mechanisms [212]. In BRAF and NRAS mutated cells, MAPK cascade is turned on without the need of ECM signaling or growth factors thereby allowing to proliferation, survival and cell transformation [213]. However, by itself oncogenic BRAF is not sufficient for melanoma and must cooperate with other processes to induce the fully cancerous state. In fact, *BRAF* is mutated in up to 80% of the benign nevi [214]. Indeed, nevi remain growth-arrested for decades and rarely progress into melanomas [215,216] presumably because aberrant BRAF signaling induces a robust senescence response mediated by upregulation of the cell cycle inhibitor p16 [217,218,219]. Escape from BRAF-induced senescence requires cooperation with other oncogenic process including additional DNA damage, epigenetic mechanisms, loss of PTEN, activation of PI3K/AKT and mTOR signaling, as well as metabolic reprogramming. In addition, microenvironmental mediators might directly and indirectly influence MAPK pathway activity in melanocyte lineage. Nevus melanocytes secrete several molecules belonging the SASP [220], a powerful autocrine/paracrine mechanism for the maintenance of the senescent state. Although *BRAF* mutation and activation of the MAPK pathway is important in nevogenesis, MAPK pathway activation do not appear to persist at high levels in nevi after growth arrest [214,215,216,217,218,219,220,221]. However, due to the potent re-activation of MAPK pathway in melanoma [222] selective BRAF inhibitors are used in the treatment of patients with BRAF-mutant advanced melanoma [223,224]. Unfortunately, patients frequently develop mutation-independent resistance to this therapy. Acquired resistance is mostly driven by the secretory activity of TAMs and MAFs [225,226,227].

Although TGF-β released locally from BRAF-inhibitor treated melanoma cells appeared to constitute an important mechanism of fibroblast activation, there is also evidence that the introduction of mutant *BRAF* into melanoma cells increases their secretion of interleukin (IL)-1α that causes tumor-associated fibroblasts to induce immune suppression [228]. In fibroblasts, the expression of mitogen-activated protein kinase kinase 1 (MAPKK1) is strongly induced by melanoma cells secretome [75]. Several studies underlined the key role of fibroblast-derived cytokines in MAPK inhibitor tolerance [225,226,227,228,229]. This effect is mostly due to the non-negligible direct effect of this class of compound on *BRAF* wild type surrounding non-melanoma cells. BRAF inhibition might lead to a paradoxical activation of MAPK in fibroblasts increasing the production of survival factor as neuregulin (NRG) and HGF [229]. Further, as an indirect effect, MAPKi-treated melanoma cells stimulate macrophages to produce IL-1β that in turn lead to the conversion of fibroblast into a melanoma-protective phenotype [225]. In a very interesting study, Hirata and collaborators demonstrated that BRAF inhibitors promote the formation of dense collagen fibrils and an overall increased matrix deposition by MAFs that render BRAF-mutant melanoma cells insensitive to treatment. Remodeled ECM leads to adhesion-dependent (integrin and focal adhesion-dependent) signaling to ERK that negates the effect of BRAF inhibition in the melanoma cells. Histological examination of melanoma sample from vemurafenib-resistant patients confirmed increased fibroblastic stroma and stiffer matrix once resistance to BRAF inhibitors had developed [230].

## 4. Conclusions

**Figure 2:**
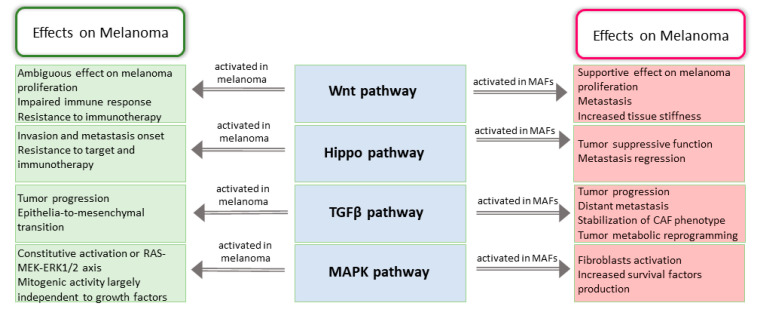
Tumor promoting function of oncogenic pathways deregulated in CAFs. Scheme recapitulates data presented concerning Wnt, Hippo, TGFβ and MAPK pathways deregulation in MAFs and melanoma cells. Mostly, activation of these pathways in MAFs exerts a pro-tumorigenic effect with the exception of Hippo signaling that trigger a competition between tumor and stroma characterized by a tumor-suppressive function.

A huge number of studies documented that the imbalance of cellular homeostasis during melanomagenesis combines oncogenic transformation of melanocytes within altered tumor stroma. Thus, from the therapeutic point of view, tumor and stroma might be considered a unique functional unit. In melanoma, most of the targetable signal transduction pathways are correspondingly altered in MAFs, suggesting that target therapies presumably deeply impact on microenvironment biological behavior. Contextual modulation of oncogenic signal cascades in MAFs might arise from extrinsic stimuli (e.g., preexisting chronic tissue damage), melanoma-induced activation and stimulation by therapeutic pressure. On the other hand, in line with symmetric bi-directional cancer-fibroblast crosstalk, the competition imposed by antitumor function of tumor microenvironment might elicit activation of intracellular signaling in tumor cells exacerbating neoplastic phenotype. ECM remodeling seems to play a central role in melanoma biology since the activation of important signal transduction pathways involved in melanomagenesis are extremely sensible to mechanotransduction. This point of view implies a shift of the therapeutic approach from the neoplastic cell-centric to a stroma-centric consideration (Figure 2). Full characterization of CAFs might be considered part of the customization of healthcare. As discussed in this review, molecules already used in clinical practice, such as MAPKi, or which are in the preclinical study phase should be reevaluated considering the intercellular molecular dialogue of neoplastic and non-neoplastic cells. In our opinion, the development of strategies able to simultaneously target melanoma cells and MAFs represents an extraordinary opportunity in the current setting of precision cancer medicine.
